# Evaluation of Four Commercial Multiplex Molecular Tests for the Diagnosis of Acute Respiratory Infections

**DOI:** 10.1371/journal.pone.0130378

**Published:** 2015-06-24

**Authors:** Nicolas Salez, Astrid Vabret, Marianne Leruez-Ville, Laurent Andreoletti, Fabrice Carrat, Fanny Renois, Xavier de Lamballerie

**Affiliations:** 1 Aix Marseille Université, IRD French Institute of Research for Development, EHESP French School of Public Health, EPV UMR_D 190 "Emergence des Pathologies Virales", Marseille, France; 2 Laboratory of Human and Molecular Virology, IFR146 ICORE, University Hospital, Caen, France; 3 Virology Laboratory, Hôpital Necker Enfants malades, APHP, Université Paris Descartes, Sorbonne Paris Cité-EA 36–20, Paris, France; 4 Unité de Virologie Médicale et Moléculaire, Centre Hospitalier Universitaire, and IFR 53/EA-4303 (DAT/PPCIDH), Faculté de Médecine, Reims, France; 5 UMR-S 707 INSERM, Pierre et Marie Curie University, Paris, France; 6 Public Assistance Hospital of Paris, Department of Internal Medicine, Hôpital Saint Antoine, Unité de Santé Publique, Paris, France; 7 IHU Institute hospitalo-universitaire Méditerranée Infection, APHM Public Hospitals of Marseille, Marseille, France; University of Hong Kong, HONG KONG

## Abstract

Acute Respiratory Infections (ARIs) are responsible for considerable morbidity and mortality worldwide. Documentation of respiratory specimens can help for an appropriate clinical management with a significant effect on the disease progress in patient, the antimicrobial therapy used and the risk of secondary spread of infection. Here, we compared the performances of four commercial multiplex kits used in French University Hospital diagnostic microbiology laboratories for the detection of ARI pathogens (*i*.*e*., the xTAG Respiratory Viral Panel Fast, RespiFinder SMART 22, CLART PneumoVir and Fast Track Diagnostics Respiratory Pathogen 33 kits). We used a standardised nucleic acids extraction protocol and a comprehensive comparative approach that mixed reference to well established real-time PCR detection techniques and analysis of convergent positive results. We tested 166 respiratory clinical samples and identified a global high degree of correlation for at least three of the techniques (xTAG, RespiFinder and FTD33). For these techniques, the highest Youden’s index (YI), positive predictive (PPV) and specificity (Sp) values were observed for Core tests (*e*.*g*., influenza A [YI:0.86–1.00; PPV:78.95–100.00; Sp:97.32–100.00] & B [YI:0.44–1.00; PPV:100.00; Sp:100.00], hRSV [YI:0.50–0.99; PPV:85.71–100.00; Sp:99.38–100.00], hMPV [YI:0.71–1.00; PPV:83.33–100.00; Sp:99.37–100.00], EV/hRV [YI:0.62–0.82; PPV:93.33–100.00; Sp:94.48–100.00], AdV [YI:1.00; PPV:100.00; Sp:100.00] and hBoV [YI:0.20–0.80; PPV:57.14–100.00; Sp:98.14–100.00]). The present study completed an overview of the multiplex techniques available for the diagnosis of acute respiratory infections.

## Introduction

Acute Respiratory Infections (ARIs) are responsible for considerable morbidity and mortality worldwide [1]. The precise and rapid documentation of respiratory specimens can lead to a significant effect on the disease progress in patients [2] when identification of the causative agents leads to providing an appropriate therapy administration, but it is also useful for decreasing the use of unnecessary antimicrobial therapy [3,4] and limiting the risk of secondary spread of infection [5]. However, ARIs are most often associated with poorly specific clinical presentations [6] and therefore, their aetiological diagnosis mainly relies on laboratory testing.

Conventional laboratory diagnostic methods have several limitations for routine detection of respiratory pathogens. For example, culture isolation of viruses of bacteria is poorly sensitive, time consuming and usually too slow to provide a biological result at the acute phase of the disease [7]; direct immunofluorescence assays and immunochromatographic antigen testing can provide results very rapidly but represent time- and labor-consuming tasks for the former, and are poorly sensitive for the latter [8,9,10]. Molecular biology testing allows the detection of a variety of viral and bacterial pathogens within hours, with excellent sensitivity and specificity and may represent a credible alternative to the aforementioned biological assays. However, given the number of aetiological agents potentially implicated in ARIs, conventional monoplex PCR assays are tedious, expensive and require large amounts of biological samples [10,11]. Consequently, multiplex molecular detection tests have been increasingly developed in recent years and a number of commercial kits have been proposed to diagnostic microbiology laboratories, which allow the detection of 12 to 33 different pathogens. This includes the xTAG Respiratory Viral Panel, Fast or v1, assays (Luminex Molecular Diagnostics, Toronto, Canada); the FilmArray respiratory viral panel assay (BioFire Diagnostics, Salt Lake City, UT); the Fast Track Diagnostics Respiratory Pathogen, 21 or 33, assays (Fast Track Diagnostics, Luxembourg); the CLART PneumoVir (Genomica, Coslada, Spain); the RespiFinder, 19 or SMART 22, assay (Pathofinder, Maastricht, Netherlands); the eSensor Respiratory Viral Panel (GenMark Dx, Carlsbad, CA); the MultiCode-PLx (EraGen Biosciences); the Easyplex respiratory pathogen 12 kit (Ausdiagnostics, Sydney, Australia); the Seeplex RV15 OneStep ACE Detection and Pneumobacter ACE Detection (Seegene Inc., Seoul, South Korea); the Magicplex RV Panel Real-time Test (Seegene Inc); and the ResPlex II Panel v2.0 (Qiagen, Hilden, Germany). Precisely evaluating such multiplex kits is difficult in the absence of undisputable golden standard reference techniques for a large number of the pathogens tested. A number of studies have compared the detection of the molecular multiplex platforms with in-house conventional molecular methods [11,12,13,14] but since the reference methods vary from study to study [15], estimating the actual performance of the assays remains difficult. More recently, direct comparison of commercialised multiplex kits was undertaken, with reference to in-house multiplex real-time PCR systems [16], or to commercial duplex PCR tests [17].

Here, as a follow-up to these pioneer studies, we compared the performances of four commercial multiplex kits used in French University Hospital diagnostic microbiology laboratories for the detection of ARI pathogens, *i*.*e*., the xTAG Respiratory Viral Panel Fast, RespiFinder SMART 22, CLART PneumoVir and Fast Track Diagnostics Respiratory Pathogen 33 kits. Our analysis mainly focussed on viral pathogens that represent the common target of all kits, the number of bacteria potentially detected varying from 4 (RespiFinder SMART 22) to 12 (Fast Track Diagnostics Respiratory Pathogen 33), making any sound comparison impossible. We combined normalised extraction of nucleic acids, "reference" monoplex real-time PCR detection and a consensus interpretation algorithm to estimate the intrinsic capability of each kit to detection of the various pathogens tested.

## Materials and Methods

### Nasopharyngeal swabs

Out of the 1,465 patients enrolled in the CoPanFlu-France cohort study [18], 166 presented with ARI during the 2010 and 2011 influenza epidemic seasons. Nasopharyngeal samples were available for all these patients and were selected for this study.

The patients' age ranged from 1 month to 79 years (median: 31.2yo).

### Broad range multiplex Kits

Four commercial kits for Multiplex detection of respiratory viruses were tested:

- The **CLART PneumoVir** (PneumoVir) assay is based on PCR-DNA micro-array detection. This assay allows the detection of 17 respiratory viruses.
- The **Fast Track Diagnostics Respiratory Pathogen 33** (FTD-RP33) version 3 assay is based on multiplex one-step reverse transcription polymerase chain reactions (RT-PCR) with probes for detecting 33 respiratory pathogens (21 viruses and 12 bacteria). It was used with the Bio-Rad CFX96 thermocycler and The SuperScript III Platinum One-Step Quantitative RT-PCR System without ROX (Invitrogen).
- The **xTAG Respiratory Viral Panel Fast** (xTagRVP-F) is based on a multiplex RT-PCR reaction in which the target-specific primers are chimeric, including a terminal Universal Tag sequence. This allows the detection of 19 different respiratory viruses [4,19] by sorting on a Luminex xMAP instrument [20].
- The **RespiFinder SMART 22** (RespiFinder) assay is based on the multiplex ligation-dependent probe amplification (MLPA) technology, preceded by a pre-amplification step [20]. Results are analysed by capillary electrophoresis [9]. This assay differentiates 18 respiratory viruses and 4 bacteria in one reaction by melt curve analysis.

The different protocols were performed according to manufacturers’ instructions but the extraction of nucleic acids was standardised to use a similar molecular substrate in each assay. One extraction dedicated to each test (each multiplex assay and for the “confirmation tests”) was performed from one of the identical 100μL aliquots prepared from nasopharyngeal swabs. The internal controls of each test were included in the dedicated extraction, and their final concentration adapted to manufacturers' recommendations. Nucleic acids were extracted using the EZ1 Virus Mini Kit v2 on the EZ1 advanced XL Biorobot workstation (Qiagen). Elution was performed in a 90μL volume and nucleic acids were subsequently stored at -80°C before analysis.

The combination of all systems potentially allowed the detection of 41 respiratory pathogens: Influenzavirus A (Inf A) (H3N2, H1N1, H5N1), Influenzavirus B (Inf B), Influenzavirus C (Inf C), Parainfluenzaviruses (PIV) (1, 2, 3 and 4a/b), human Respiratory Syncytial Virus (hRSV) (A and B), Enteroviruses (EV), Rhinoviruses (hRV), Adenoviruses (AdV), Parechoviruses, human Bocavirus (hBoV), human Coronaviruses (hCoV) (229E, NL63, OC43, HKU1), human Metapneumovirus (hMPV) (A and B), Cytomegalovirus (CMV), *Pneumocystis jirovecii* (PCP), *Mycoplasma pneumoniae* (Mpneu), *Chlamydia pneumoniae* (Cpneu), *Streptococcus pneumoniae* (Spneu), *Haemophilus influenzae species* (Haeinf), *Haemophilus influenzae type B* (HIB), *Staphylococcus aureus* (Saur), *Moraxella catarrhalis* (Morax), *Bordetella pertusis* (Bord), *Klebsiella pneumoniae* (Kpneu), *Legionella species* (Legio) and *Salmonella species* (Salm).

The spectrum of detection of the different kits is reported in Table 1. Tests allowing the detection of respiratory pathogens were classified in two different classes: the "Core tests" and the "Extra tests", the "Core tests" being defined as those corresponding to pathogens detected by all four kits.

**Table 1:** Respiratory pathogens detected by each Kit.

Viruses					Bacteria				
	PneumoVir	FTD-RP33	xTagRVP-F	RespiFinder		PneumoVir	FTD-RP33	xTagRVP-F	RespiFinder
**Inf A**	√	√		√	**Pneumo jiro**		√		
**H1N1**	√		√		**Myco pneumo**		√		√
**H1N1pdm09**	√			√	**Chlam pneumo**		√		√
**H3N2**	√		√		**Strepto pneumo**		√		
**Inf B**	√	√	√	√	**Haemo inf sp**		√		
**Inf C**	√	√			**Haemo inf B**		√		
**PIV 1**	√	√	√	√	**Staph aur**		√		
**PIV 2**	√	√	√	√	**Morax catarr**		√		
**PIV 3**	√	√	√	√	**Bord pert**		√		√
**PIV 4**		√	√	√	**Klebs pneumo**		√		
**PIV 4 a**	√				**Legio sp**		√		√
**PIV 4 b**	√				**Salmo sp**		√		
**hRSV**		√							
**hRSV A**	√		√	√					
**hRSV B**	√		√	√					
**EV/ hRV**			√	√					
**hRV**	√	√							
**EV**	√	√							
**AdV**	√	√	√	√					
**EVs**	√	√	√	√					
**hPEV**		√							
**hBoV**	√	√	√	√					
**hCoV 229E**	√	√	√	√					
**hCoV NL63**		√	√	√					
**hCoV OC43**		√	√	√					
**hCoV HKU1**		√	√	√					
**hMPV**	√	√	√	√					
**hMPV A**	√								
**hMPV B**	√								
**CMV**		√							

Inf A, B, C: Influenza A, B, C; H1N1: Influenza A H1N1; H1N1pdm09: Influenza A H1N1pdm 2009; H3N2: Influenza A H3N2; PIV 1, 2, 3, 4, 4a, 4b: Parainfluenzavirus 1, 2, 3, 4, 4a, 4b; hRSV: human Respiratory Syncytial Virus; hRSV A, B: human Respiratory Syncytial Virus A, B; EV: Enterovirus; hRV: human Rhinovirus; AdV: Adenovirus; hBoV: human Bocavirus; hCoV 229E, OC43, NL63, HKU1: human Coronavirus type 229, OC43, NL63, HKU1; hMPV: human Metapneumovirus; hMPV A, B: human Metapneumovirus A, B; CMV: Cytomegalovirus; EVs: Echovirus; hPeV: human Parechovirus; Pneumo jiro: *Pneumocystis jirovecii;* Myco pneumo: *Mycoplasma pneumoniae;* Chlam pneumo: *Chlamydia pneumoniae;* Strepto pneumo: *Streptococcus pneumoniae;* Haemo inf sp: *Haemophilus influenzae* species; Haemo inf B: *Haemophilus influenzae* type B; Staph aur: *Staphylococcus aureus;* Morax catarr: *Moraxella catarrhalis;* Bord pert: *Bordetella pertussis;* Klebs pneumo: *Klebsiella pneumoniae;* Legio sp: *Legionella* species; Salmo sp: *Salmonella* species; PneumoVir: CLART PneumoVir; FTD-RP33: Fast Track Diagnostics Respiratory Pathogen 33; xTagRVP-F: xTAG Respiratory Viral Panel Fast; RespiFinder: RespiFinder SMART 22

### Processing of samples and interpretation of results

**Fig 1:**
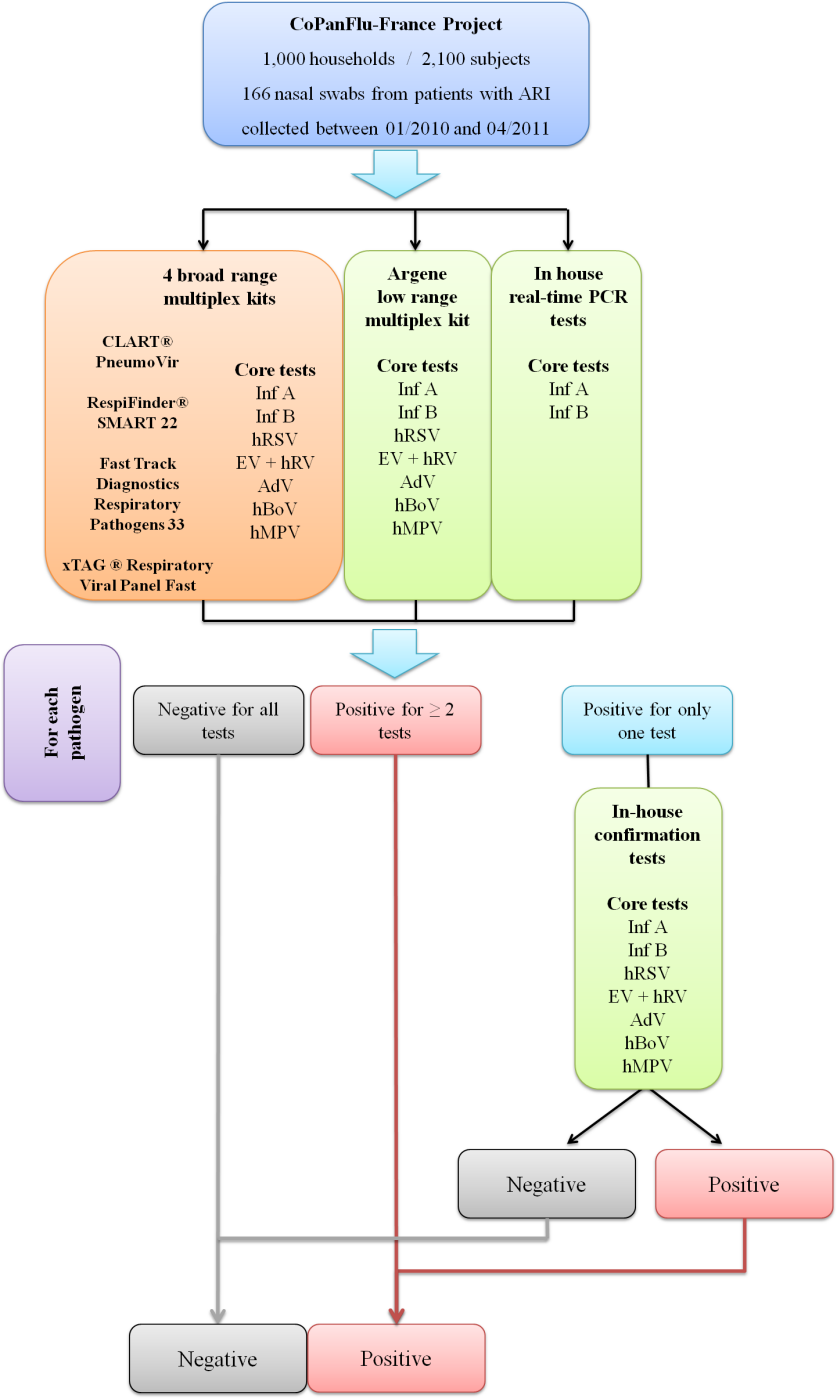
Decision tree (algorithm used for Core tests). (Inf A: Influenza A, Inf B: Influenza B, hRSV: human Respiratory Syncytial Virus, hMPV: human Metapneumovirus, EV + hRV:; Enterovirus + human Rhinovirus, AdV: Adenovirus and hBoV: human Bocavirus).

Firstly, all samples were tested with the four broad range multiplex kits (Fig 1). In addition, they were systematically tested for Inf A and Inf B using in-house standard real-time RT-PCR assays [21], and for Core tests (Inf A, Inf B, hRSV, hMPV, EV + hRV, AdV and hBoV) using the Argene (Respiratory Multi Well System r-gene) low range kit, consisting of 4 Taqman-based duplex PCR or RT-PCR reactions (Inf A+B, hRSV+hMPV, hRV&EV+cellular control, AdV+hBoV).

Samples testing negative for a given pathogen with all detection methods were proposed negative for this pathogen. Samples testing positive for a pathogen with at least 2 different techniques were proposed positive. Samples with discrepant results (positive for one pathogen with only one detection method) were further processed with "confirmation" assays, consisting of one or several real-time or nested amplification protocols (Table A in S1 File) and subsequently declared positive or negative according to the result obtained.

Positive predictive value (PPV), negative predictive value (NPV), Sensitivity, Specificity and agreement (as evaluated by the Youden’s index) values were calculated for the Core tests of the kits tested (see below).

Secondly, all samples were analysed according to the results obtained by the four broad range multiplex kits for Extra tests (S1 Fig): Influenza A H1N1pdm 2009; "seasonal" Influenza A H1N1; Flu A H5N1; Flu A H3N2; hRSV A/B, hMPV A/B, PIV 1/2/3/4a/4b, PCP and bacteria (Mpneu, Cpneu, Spneu, Haeinf, HIB, Saur, Morax, Bord, Kpneu, Legio and Salm).

Only results relating to viral pathogens were analysed in depth since a majority of bacterial pathogens were detected only by the FTD-RP33 test.

A decision tree similar to that used for Core tests was designed and confirmation techniques were used to confirm or invalidate discrepant results (Table B in S1 File). PPV, Sensitivity, NPV, and Specificity values were calculated for Extra tests (see below).

### Performances of the kits

To compare the performances of the kits, Sensitivity, Specificity and Positive and Negative Predictive values were calculated. In accordance with the interpretation rules decreed in the previous section, calculations relied on the following definitions: ***(i)*** True Positives were Positive results confirmed by another kit or a confirmation test; ***(ii)*** False Positives were Positive results that were not confirmed by another kit or a confirmation test; ***(iii)*** False Negatives were Negative results invalidated by at least 2 other kits or 1 kit and 1 confirmation test; ***(iv)*** True Negatives were Negative results that were not invalidated by at least 2 other kits or 1 kit and 1 confirmation test.

### Virus positive controls and limits of detection

Culture supernatants were obtained from the European virus archive collection (EVA, www.europeanvirusarchive.com) for Influenza A H1N1 (A/Marseille/9410380/2009), Influenza A H3N2 (A/Marseille/4781598/2009), Influenza B (B/Marseille/4461097/2008), Influenza C (C/Leningrad:232/83), Respiratory Syncytial virus type A (Long) and B (Gb2), Rhinovirus (MAR2007 3813047), Enterovirus (Human Coxsackievirus B3 clinical strain B32679), human Parechovirus type 3 (UNK), Adenovirus type B (MAR2007 7416184 clinical strain). In the case of the human Metapneumovirus type A, a sample testing positive using both molecular detection and direct immunofluorescence was used as a positive control.

Nucleic acids were extracted as described above but using an initial sample volume of 400μL and an elution volume of 120μL. Tenfold serial dilutions of nucleic acids were performed and tested using the relevant in-house "confirmation" real time PCR or RT-PCR assays (S1 File). The last three dilutions allowing unambiguous detection (*i*.*e*., the lowest dilution had a Ct at 33 and the highest dilution had a Ct between 37 and 40) were used to create a panel, which was subsequently used to evaluate the limit of detection of the 4 broad range multiplex kits.

### Ethics statement

The CoPanFlu-France cohort protocol was approved by the French ethics committee (“Comité de Protection des Personnes Ile-de-France I”) on 8 September 2009. All study participants provided written informed consent [18].

## Results

The analysis of 166 Nasopharyngeal samples using a combination of four commercial broad range multiplex kits and of confirmation tests provided confirmed aetiological viral diagnosis for 87 samples (52%), including 8 samples (4.8%) with confirmed dual infection (see details in Table 2).

**Table 2:** Viral etiologies identified and bacterial results.

Viruses (Confirmed results)	Nb
**hRV**	27
**EV**	1
**EV/RV** *	6
**H1N1pdm09**	17
**Inf B**	9
**hBoV**	5
**hCoV HKUI**	4
**hCoV NL63**	4
**hCoV OC43**	4
**hCoV 229E**	1
**hMPV A**	4
**hMPV B**	3
**hRSV A**	4
**hRSV B**	2
**PIV 2**	2
**PIV 1**	1
**AdV**	1
**Total number of virus detected**	95
**EV/hRV+ hBoV**	1
**H1N1pdm09 + RV**	1
**Inf B + hBoV**	1
**Inf B + RV**	1
**PIV 1 + hCoV HKUI**	1
**EV/hRV+ hMPV A**	1
**hRSV A + hMPV A**	1
**hRSV B+ AdV**	1
**Total number of viral dual infections**	8
**Total number of viral positive samples**	87
**Percentage of viral positive samples (n = 166)**	52.40%
**Bacteria (Not confirmed results)** **	Nb
**Total number of bacteria detected**	104
**Total number of bacterial dual infections**	14
**Total number of bacterial co-infections (at least 2 other bacteria)**	6
**Total number of bacteria positive samples**	77
**Percentage of bacteria positive samples (n = 166)**	25.30%
**Total number of co-infections (Virus/bacteria)**	40
**Total number of positive samples (confirmed for viruses but not for bacteria)**	124
**Percentage of positive samples (n = 166)**	74.70%

Inf A, B: Influenza A, B; H1N1: Influenza A H1N1; H1N1pdm09: Influenza A H1N1pdm 2009; PIV 1, 2: Parainfluenzavirus 1, 2; hRSV A, B: human Respiratory Syncytial Virus A, B; EV: Enterovirus; hRV: human Rhinovirus; AdV: Adenovirus; hBoV: human Bocavirus; hCoV 229, OC43, NL63, HKU1: human Coronavirus type 229, OC43, NL63, HKU1; hMPV A, B: human Metapneumovirus A, B.

(*) EV/ hRV detected no determined for Enterovirus or human Rhinovirus

(**) Bacteria etiologies identified are shown in supplementary information (S1 Table)

The distribution of confirmed positive detections provided the following results: Enterovirus/Rhinovirus: 34 (39.1%); Influenza A H1N1 2009: 17 (19.5%); Coronavirus: 13 (14.9%); Influenza B: 9 (10%); Metapneumovirus: 7 (8.1%); Respiratory Syncytial Virus: 6 (6.9%); Bocavirus: 5 (5.7%); Parainfluenzaviruses: 3 (3.5%); Adenovirus: 1 (1.1%).

### Performances of the four commercial broad range multiplex kits

The performances of the kits for the detection of specific pathogens are presented in Table 3. Regarding the detection of Influenza A viruse(s), the sensitivity and PP values ranged from 88.2% and 79% respectively for the PneumoVir kit to 100% for the FTD-RP33 kit. Regarding Influenza B, the best sensitivity was observed for the RespiFinder kit (100%). It was under 80% for the PneumoVir and FTD-RP33 kits and under 50% for the xTagRVP-F kit. PP values were at 100% for all kits except the PneumoVir kit (87.5%).

**Table 3:** Performances of the four kits for individual Core tests (Inf A, Inf B, hRSV, hMPV, EV + hRV, AdV and hBoV), parainfluenza viruses, Influenza A H1N1pdm 2009 and coronaviruses.

**CLART PneumoVir** ^**a**^														
	**Inf A**	**H1N1pdm09**	**Inf B**	**PIV**	**hRSV**	**EV/hRV**	**AdV**	**hBoV**	**hCoV**	**hCoV 229E**	**hCoV NL63**	**hCoV OC43**	**hCoV HKU1**	**hMPV**
**Positives**	19	17	8	3	5	15	2	3	0	1	0	0	0	9
**True Positives**	15	14	7	2	4	14	1	2	0	1	0	0	0	7
**False Positives**	4	3	1	1	1	1	1	1	0	0	0	0	0	2
**True Negatives**	145	147	156	162	159	131	164	160	153	165	162	162	162	157
**False Negatives**	2	2	2	1	2	20	0	3	13	0	4	4	4	0
**PPV**	78.95%	82.35%	87.50%	66.67%	80.00%	93.33%	50.00%	66.67%	-	100.00%	-	-	-	77.78%
**Sensitivity**	88.24%	87.50%	77.78%	66.67%	66.67%	41.18%	100.00%	40.00%	-	100.00%	-	-	-	100.00%
**NPV**	98.64%	98.66%	98.73%	99.39%	98.76%	86.75%	100.00%	98.16%	-	100.00%	-	-	-	100.00%
**Specificity**	97.32%	98.00%	99.36%	99.39%	99.38%	99.24%	99.39%	99.38%	-	100.00%	-	-	-	98.74%
**Youden's index**	0.86	0.86	0.77	0.66	0.66	0.40	0.99	0.39	-	1.00	-	-	-	0.99
**Fast Track Diagnostics Respiratory Pathogen 33** ^**b**^														
	**Inf A**	**H1N1pdm09**	**Inf B**	**PIV**	**hRSV**	**EV/hRV**	**AdV**	**hBoV**	**hCoV**	**hCoV 229E**	**hCoV NL63**	**hCoV OC43**	**hCoV HKU1**	**hMPV**
**Positives**	17	0	7	2	3	21	1	7	13	2	4	4	3	7
**True Positives**	17	0	7	2	3	21	1	4	12	1	4	4	3	7
**False Positives**	0	0	0	0	0	0	0	3	1	1	0	0	0	0
**True Negatives**	149	150	157	163	160	132	165	158	152	164	162	162	162	159
**False Negatives**	0	16	2	1	3	13	0	1	1	0	0	0	1	0
**PPV**	100.00%	-	100.00%	100.00%	100.00%	100.00%	100.00%	57.14%	92.31%	50.00%	100.00%	100.00%	100.00%	100.00%
**Sensitivity**	100.00%	-	77.78%	66.67%	50.00%	61.76%	100.00%	80.00%	92.31%	100.00%	100.00%	100.00%	75.00%	100.00%
**NPV**	100.00%	-	98.74%	99.39%	98.16%	91.03%	100.00%	99.37%	99.35%	100.00%	100.00%	100.00%	99.39%	100.00%
**Specificity**	100.00%	-	100.00%	100.00%	100.00%	100.00%	100.00%	98.14%	99.35%	99.39%	100.00%	100.00%	100.00%	100.00%
**Youden's index**	1.00	-	0.78	0.67	0.50	0.62	1.00	0.78	0.92	0.99	1.00	1.00	0.75	1.00
**xTAG Respiratory Viral Panel Fast**														
	**Inf A**	**H1N1pdm**	**Inf B**	**PIV**	**hRSV**	**EV/hRV**	**AdV**	**hBoV**	**hCoV**	**hCoV 229E**	**hCoV NL63**	**hCoV OC43**	**hCoV HKU1**	**hMPV**
**Positives**	19	19	4	2	4	30	0	1	9	1	4	3	1	6
**True Positives**	15	15	4	2	4	28	0	1	9	1	4	3	1	6
**False Positives**	4	4	0	0	0	2	0	0	0	0	0	0	0	0
**True Negatives**	145	146	157	163	160	130	165	161	153	165	162	162	162	159
**False Negatives**	2	1	5	1	2	6	1	4	4	0	0	1	3	1
**PPV**	78.95%	78.95%	100.00%	100.00%	100.00%	93.33%	-	100.00%	100.00%	100.00%	100.00%	100.00%	100.00%	100.00%
**Sensitivity**	88.24%	93.75%	44.44%	66.67%	66.67%	82.35%	0.00%	20.00%	69.23%	100.00%	100.00%	75.00%	25.00%	85.71%
**NPV**	98.64%	99.32%	96.91%	99.39%	98.77%	95.59%	99.40%	97.58%	97.45%	100.00%	100.00%	99.39%	98.18%	99.38%
**Specificity**	97.32%	97.33%	100.00%	100.00%	100.00%	98.48%	100.00%	100.00%	100.00%	100.00%	100.00%	100.00%	100.00%	100.00%
**Youden's index**	0.86	0.91	0.44	0.67	0.67	0.81	-	0.20	0.69	1.00	1.00	0.75	0.25	0.86
**RespiFinder SMART 22**														
	**Inf A**	**H1N1pdm**	**Inf B**	**PIV**	**hRSV**	**EV/hRV**	**AdV**	**hBoV**	**hCoV**	**hCoV 229E**	**hCoV NL63**	**hCoV OC43**	**hCoV HKU1**	**hMPV**
**Positives**	17	13	9	3	7	29	1	4	15	1	4	6	4	6
**True Positives**	16	13	9	2	6	28	1	4	13	1	4	4	4	5
**False Positives**	1	0	0	1	1	1	0	0	2	0	0	2	0	1
**True Negatives**	148	150	157	162	159	131	165	161	151	165	162	160	162	158
**False Negatives**	1	3	0	1	0	6	0	1	0	0	0	0	0	2
**PPV**	94.12%	100.00%	100.00%	66.67%	85.71%	96.55%	100.00%	100.00%	86.67%	100.00%	100.00%	66.67%	100.00%	83.33%
**Sensitivity**	94.12%	81.25%	100.00%	66.67%	100.00%	82.35%	100.00%	80.00%	100.00%	100.00%	100.00%	100.00%	100.00%	71.43%
**NPV**	99.33%	98.04%	100.00%	99.39%	100.00%	95.62%	100.00%	99.38%	100.00%	100.00%	100.00%	100.00%	100.00%	98.75%
**Specificity**	99.33%	100.00%	100.00%	99.39%	99.38%	99.24%	100.00%	100.00%	98.69%	100.00%	100.00%	98.77%	100.00%	99.37%
**Youden's index**	0.93	0.81	1.00	0.66	0.99	0.82	1.00	0.80	0.99	1.00	1.00	0.99	1.00	0.71

Criteria for the definition of true positives, false positives, true negatives and false negatives are provided in the 'Materials and Methods' section, 'Performances of the kits' sub-section.

^a^ Kit having only probe for the detection of Coronavirus E229, and not for HKU1, OC43 and NL63.

^b^ Kit having probe for the detection all Influenza A viruses and not for specific detection of H1N1 2009 pdm.

Globally, the performances of the PneumoVir kit were hindered by the existence of false positive results for a proportion of the pathogens tested, but also of false negative results for the most common pathogens and in particular human Rhinoviruses/Enteroviruses. The performances of the xTagRVP-F kit were hampered by its low sensitivity for the detection of Influenza B, human bocaviruses and human coronaviruses, but the kit was associated with high PP values (very few false positives). This profile is somewhat similar to that of the FTD-RP33 kit that exhibited high PP values and perfectible sensitivity for the detection of Influenza B, Parainfluenzaviruses, human Respiratory Syncytial virus and human Rhinoviruses/ Enteroviruses. The RespiFinder Kit displayed the highest overall sensitivity values, associated with high PP values. Its lowest performances were for human Metapneumoviruses (sensitivity: 71.4%) and Parainfluenzaviruses (66.6%, similar with other kits).

Regarding the global performances of the kits, the Positive Predictive values were >90% for three of the four kits (xTagRVP-F, FTD-RP33 and RespiFinder) (Table 4). The highest overall specificity was observed for the FTD-RP33 kit (94.4%), with a 100% score for five common pathogens (Influenzaviruses A and B, Parainfluenzaviruses, human Respiratory Syncytial viruses, human Rhinoviruses/Enteroviruses). The most important differences between the kits tested were identified for the overall sensitivity (ranging from 63.9% for the PneumoVir Kit to 88.4% for the RespiFinder Kit) and for the Negative Predictive Value (ranging from 70% for the PneumoVir Kit to 85.3% for the RespiFinder Kit).

The Youden’s index, which summarise the performance of a diagnostic test, was globally strong (> 0.60) except for the PneumoVir kit (0.48).

**Table 4:** Global comparison of PPV, Sensitivity, NPV, Specificity and Youden's index values between kits for all tests.

	PneumoVir	xTagRVP-F	FTD-RP33	RespiFinder
**Number of true positives (TP)**	53	69	74	84
**Number of false positives (FP)**	13	6	4	7
**Number of true negatives (TN)**	70	65	67	64
**Number of false negatives (FN)**	30	26	21	11
**PPV**	80.30%	92.00%	94.87%	92.31%
**Sensitivity**	63.86%	72.63%	77.89%	88.42%
**NPV**	70.00%	71.43%	76.14%	85.33%
**Specificity**	84.34%	91.55%	94.37%	90.14%
**Youden's index**	0.48	0.64	0.72	0.79

Criteria for the definition of true positives, false positives, true negatives and false negatives are provided in the 'Materials and Methods section', 'Performances of the kits' sub-section. PneumoVir: CLART PneumoVir; xTagRVP-F: xTAG Respiratory Viral Panel Fast; FTD-RP33: Fast Track Diagnostics Respiratory Pathogen 33; RespiFinder: RespiFinder SMART 22

### Limits of detection of the four commercial broad range multiplex kits

**Fig 2:**
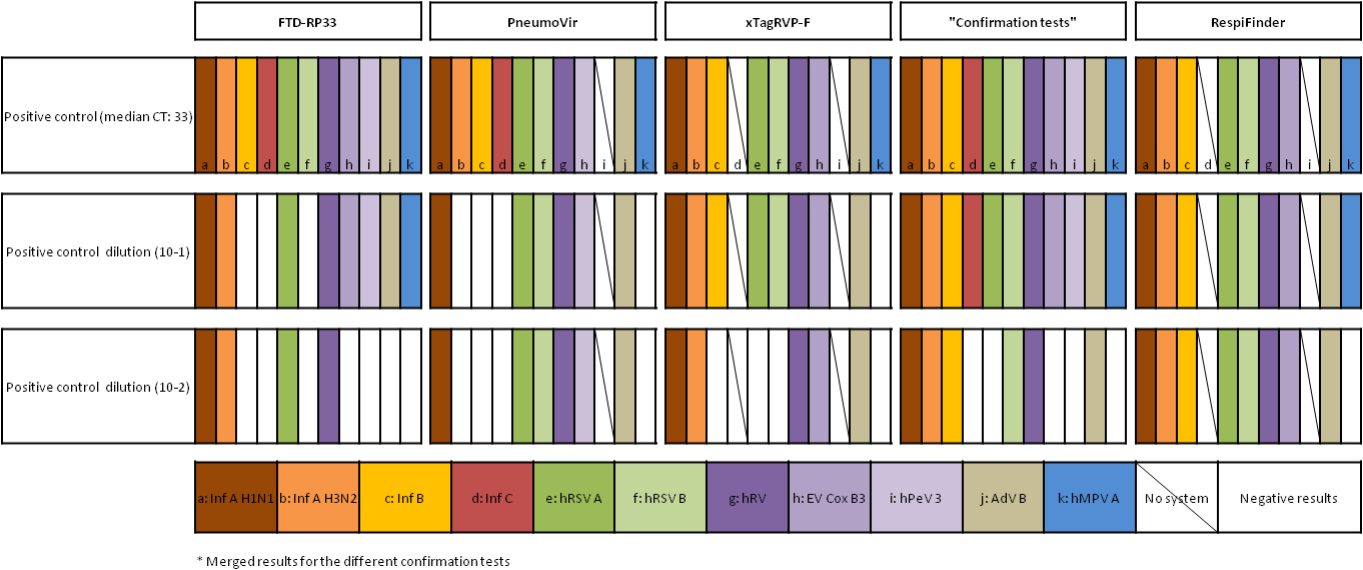
Estimate of detection limit of the different kits. Inf A H1N1: Influenza A H1N1 (A/Marseille/9410380/2009), Inf A H3N2: Influenza A H3N2 (A/Marseille/4781598/2009), Inf B: Influenza B (B/Marseille/4461097/2008), Inf C: Influenza C (C/Leningrad:232/83), hRSV A and B: human Respiratory Syncytial virus type A (Long) and B (Gb2), hRV: Rhinovirus (MAR2007 3813047), EV Cox B3: Enterovirus (Human Coxsackievirus B3 clinical strain B32679), hPeV: human Parechovirus type 3 (UNK), AdV B: Adenovirus type B (MAR2007 7416184 clinical strain), and hMPV A: human Metapneumovirus type A.

Results are presented in Fig 2 and show that, at the highest dilution proposed, the RespiFinder kit detected 8 pathogens (out of the 9 tested), whilst the PneumoVir kits detected 6 (out of 10 tested), the xTagRVP-F kits detected 5 (out of 9 tested) and the FTD-RP33 detected 4 (out of 11 tested).

## Discussion

We have compared 4 commercialised multiplex PCR techniques for the detection of respiratory pathogens using standardised nucleic acid extracts from French patients presenting with ARIs in 2010 and 2011. Our study mainly focussed on viral pathogens.

There is previous significant information relating to the comparison of the performances of the kits studied with conventional diagnostic technique. This includes: *(i)* direct immunofluorescence, viral culture, and home-brew PCR in the case of the CLART PneumoVir assay [22]; *(ii)* home-brew PCR, direct immunofluorescence, and viral culture in the case of the xTAG Respiratory Viral Panel Fast assay [7,19,23–25]; *(iii)* home-brew PCR, and in-house multiplex real-time PCR in the case of the Fast Track Diagnostics Respiratory Pathogen assay [13,16]; *(iv)* home-brew PCR and viral culture in the case of the RespiFinder SMART 22 assay [26,27]. In addition, previous studies included comparison with other multiplex commercial kits: xTAG Respiratory Viral Panel Fast assay *versus* FilmArray respiratory viral panel, ResPlex II Panel v2.0, MultiCode-PLx, Seeplex RV15 OneStep ACE Detection and Pneumobacter ACE Detection and xTAG Respiratory Viral Panel v1 kits [7,23,25]; RespiFinder SMART 22 assay *versus* Seeplex RV15 OneStep ACE Detection and Pneumobacter ACE Detection, ResPlex II Panel v2.0, RespiFinder 19 kits [2,9,26,27]; Fast Track Diagnostics Respiratory Pathogen 21 *versus* xTAG Respiratory Viral Panel fast and Easyplex respiratory pathogen 12 kits [16]. The most complete study compared six commercialised techniques, *i*.*e*., the RespiFinder SMART 22, Seeplex RV15 OneStep ACE Detection and Pneumobacter ACE Detection, Magicplex RV Panel Real-time Test, CLART PneumoVir, xTAG Respiratory Viral Panel fast and ResPlex II Panel v2.0 kits [17].

Globally, the literature indicates that multiplex molecular techniques compare favourably with traditional diagnostic technique. They can be (at least partially) automated, are highly reproducible and are generally more sensitive.

However, **a first difficult issue for comparing respiratory pathogens multiplex techniques** is the absence of gold standard for the detection of respiratory pathogens. If we refer only to the ability of a technique to detect a pathogen or some of its molecular components in biological samples, some newly developed molecular techniques may be more sensitive than the reference techniques, (*i*.*e*., some "false positives", referring to the reference technique, may be true positives that have not been detected by this reference method). This situation may be met when the new technique is able to detect viral variants that could not be detected by previous methods and, in that case, corresponds to an actual improvement of the technique if the variants detected are true pathogens that can be implicated in ARIs. However, it can also be met when the new technique is able to detect a lower viral load. The actual clinical impact of an improved detection of low viral loads in respiratory samples has not been accurately evaluated and its medical significance at the acute phase of ARIs obviously requires to be further documented. Altogether, this indicates that the interpretation of evaluation scores should remain careful and open-minded. Accordingly, we have tried to build a comprehensive approach of the comparison of diagnostic kits that mixed reference to well established–and clinically evaluated- real-time PCR detection techniques (this included in the current study the Argene Respiratory Multi Well System r-gene low range kit for Core tests, which has been used for years for routine detection in medical virology laboratories; and a series of published home-brewed techniques) and analysis of convergent positive results. For the latter, we considered that, given *(i)* the intrinsic high specificity of molecular amplification techniques, and *(ii)* the fact that the techniques compared used different technologies and amplification primers, the probability of obtaining false positive results with two different techniques for the same sample was negligible for an evaluation purpose. Consequently, samples in which a given pathogen was detected independently by two different techniques were declared positive for this pathogen, otherwise it was declared negative. We cannot exclude that a few samples were wrongly assigned a negative result (if a specific technique performed better than all others for a given pathogen), but the high degree of convergence between the different techniques suggests that the number of "false negative" results that could have been potentially generated is limited.

**A second difficult issue for comparing respiratory pathogens multiplex techniques** is the choice of the panel of samples tested. The nature and frequency of pathogens present in the samples tested are highly dependent on the epidemiological circumstances and on the populations tested, and have an obvious influence on the results of the analysis, *e*.*g*., the low number or absence of positive samples for some viruses represents in most of the studies an obvious obstacle for accurate comparisons. In the current study, we tested patients with ARIs from all age groups recruited in a household cohort that aimed at providing a picture of the French general population. This means that, in comparison with previous hospital-based studies, the paediatric population was under-represented and the clinical presentations were milder. In addition, the specific period studied (2010–2011) was associated with the predominant circulation of the pandemic H1N1 variant of the influenza A virus. Accordingly, the low number or absence of parainfluenza virus, human coronavirus type 229E, H3N2 influenza A virus, influenza C virus, human adenovirus and human parechovirus infections did not allow a significant comparison of kits for these pathogens. This is important to notice since in previous studies the low number of positive samples for some pathogens may have concealed the poor performances of some kits for detecting these pathogens (*e*.*g*., the case of Adenoviruses for the xTAG Respiratory Viral Panel fast assay [9,17,25]).

**A third difficult issue for comparing respiratory pathogens multiplex techniques** relies in the different spectra of detection and precision of identification offered by the various commercialised kits. To take this aspect into account, we have identified "Core tests", *i*.*e*., a series of common pathogens detected by all kits (Inf A, Inf B, hRSV, hMPV, EV + hRV, AdV and hBoV). We have also limited our analysis to viral pathogens since bacterium detection was absent or very limited in 3 of the 4 kits tested. The simultaneous detection of bacteria and viruses represents a theoretical great advantage in terms of clinical management (*e*.*g*., for determining the appropriateness of starting an antibiotic treatment). However, it is noticeable that, in the current study, bacteria detection provided a large number of positive results, potentially shifting the rate of aetiological elucidation from 52% to >77% in the panel studied. Obviously, this deserves further in depth investigations, which should include samples from both ARIs and paired asymptomatic individuals. Determining the actual imputability of bacterial detection in the occurrence of ARIs may be difficult to achieve from qualitative results and clinically relevant interpretation algorithms remain to be established. As noted above, the detection of low pathogen loads may be an issue for viruses, but the case of bacteria is specifically difficult due to the frequent asymptomatic carriage of potentially pathogenic bacteria.

For Core tests, we identified an important degree of correlation between the performance of the xTAG Respiratory Viral Panel fast, the Fast Track Diagnostics Respiratory Pathogen 33 and the RespiFinder SMART 22 assays (Youden’s index: 0.67; 0.73 and 0.83, respectively) (Table 5). The CLART PneumoVir assay exhibited the most divergent performance (global Core tests Youden’s index value: 0.51; global Core tests sensitivity and specificity values: 63.3% and 87.4%, respectively), despite previous favourable performance by comparison with home-brew PCR techniques [22]. When examined in more details (Table 3), the sensitivity values for Core tests compared with those of the other kits, except for human Rhinoviruses/Enteroviruses (sensitivity: 41.2%) and human Bocaviruses (40%). There was also a trend for the existence of false positive results for Influenza A and Metapneumovirus tests, which altered the global positive predictive value of the kit (81.9% *vs* >90% for all other kits). False positive results may hypothetically be related to the fact that the technique includes an open-tube post amplification step, potentially associated with an increased risk of PCR contamination. In serial dilution tests, 4 (Inf A H3N2, Inf B, Inf C and hMPV A) out of the 10 pathogens detected in the most concentrated dilution tested negative at dilutions 10^−1^ and 10^−2^ (Fig 2).

**Table 5:** Global comparison of PPV, Sensitivity, NPV, Specificity and Youden's index values between kits for Core tests (Inf A, Inf B, hRSV, hMPV, EV + hRV, AdV and hBoV).

	PneumoVir	xTagRVP-F	FTD-RP33	RespiFinder
**Number of true positives (TP)**	50	58	60	69
**Number of false positives (FP)**	11	6	3	4
**Number of true negatives (TN)**	76	81	84	83
**Number of false negatives (FN)**	29	21	19	10
**PPV**	81.97%	90.63%	95.24%	94.52%
**Sensitivity**	63.29%	73.42%	75.95%	87.34%
**NPV**	72.38%	79.41%	81.55%	89.25%
**Specificity**	87.36%	93.10%	96.55%	95.40%
**Youden's index**	0.51	0.67	0.73	0.83

Criteria for the definition of true positives, false positives, true negatives and false negatives are provided in the 'Materials and Methods section', 'Performances of the kits' sub-section.PneumoVir: CLART PneumoVir; xTagRVP-F: xTAG Respiratory Viral Panel Fast; FTD-RP33: Fast Track Diagnostics Respiratory Pathogen 33; RespiFinder: RespiFinder SMART 22

Our study globally confirms the previously observed good performances of the xTAG Respiratory Viral Panel Fast [2,9,7,15,17,23–25,28]. For Core tests, the global sensitivity and specificity values were 73.4% and 93.1%, respectively (Table 5). The poorest results were observed for influenza A positive predictive value (78.9%) and influenza B and human bocavirus sensitivity values (44.4% and 20%, respectively). In serial dilution tests, out of the 9 pathogens detected in the most concentrated dilution, one (hMPV A) tested negative at dilution 10^−1^ and 4 (Inf B, hRSV A, hRSV B and hMPV A) at dilution 10^−2^.

The Fast Track Diagnostics Respiratory pathogens 33 kit performed well for Core tests (global sensitivity and specificity values were 75.9% and 96.5%) (Table 5). The excellent specificity values (the highest in the panel tested) may be related in part to the simple and safe real-time PCR format of the technique, which does not require any post-PCR manipulation. Amongst Core tests, and in agreement with previous findings [13], the lowest sensitivity values were observed for human Respiratory Syncytial Virus and Enterovirus/Rhinovirus (50% and 61.8%, respectively). In serial dilution tests, out of the 11 pathogens detected in the most concentrated dilution, 3 (Inf B and C, hRSV B) tested negative at dilution 10^−1^ and 7 (Inf B and C, hRSV B, EV Cox B3, hPeV, hAdV B and hMPV A) at dilution 10^−2^, in coherence with previous observations [13].

The RespiFinder SMART 22 assay, provided for Core tests both the highest Youden’s index performance values (global Core tests Youden’s index value: 0.83) and the best global sensitivity (87.3%). It also was associated with an excellent specificity (95.4%), in agreement with previous evaluations [2,9,26,27]. Amongst Core tests, the lowest sensitivity value was observed for human Metapneumovirus (71.4%). The kit also provided the best results in serial dilution tests: of the 9 pathogens detected in the most concentrated dilution, all tested positive at dilution 10^−1^ and 1 (hMPV A) tested negative at dilution 10^−2^.

## Conclusion

The present study identified a high degree of correlation for at least three of the techniques and completed an overview of the multiplex techniques available for the diagnosis of acute respiratory infections. All of the kits evaluated performed properly for Core tests and Youden’s index, positive predictive and specificity values were systematically higher for Core tests than for Extra tests. Significant differences in the ability of the kits to detect low viral loads were identified, but the medical implications of this observation remain to be evaluated. The range of detected pathogens, the technology used for PCR product revelation, the laboratory organization and other issues, such as sample through-put, staffing levels and staff expertise, clinicians’ expectations and overall funding structures appear as determinant features for the selection of a kit.

## Supporting Information

S1 File PCR confirmation assays for Core tests (Table A) and extra-tests (Table B). (DOCX)

S1 Table Bacterial etiologies identified, co-infection with viruses and viral results. (DOCX)

S1 Fig Decision tree (algorithm used for extra-tests). (TIFF)
