# Supplementary material for: Evaluation of Four Commercial Multiplex Molecular Tests for the Diagnosis of Acute Respiratory Infections
Source: PLoS One. 2015 Jun 24;10(6):e0130378. doi: 10.1371/journal.pone.0130378 (PMC4481272; doi:10.1371/journal.pone.0130378)
Supplement: S1 File — (DOCX) [file pone.0130378.s001.docx]

**File S1. PCR confirmation assays for Core tests (Table A) and extra-tests (Table B).**

Core tests (Table A)

| **Virus** | **Standard PCR** | **PCR with SYBR-Green** | **PCR with probe** | | **NESTED PCR** |
| --- | --- | --- | --- | --- | --- |
| **human Bocavirus** |  |  | [1] |  |  |
| **Adenovirus** |  |  | [2,3] |  |  |
| **human Metapneumovirus A and B** |  |  | Home-made |  |  |
| **human Respiratory Syntitial virus A and B** |  |  | [4] |  | [5,6] |
| **human Rhinovirus** |  |  | Home-made | [7] |  |
| **Enterovirus** |  |  | [7,8,9] |  |  |
| **Influenzavirus A** |  | [10] | [10] |  | [11] |
| **Influenzavirus B** |  | [10] | [10] |  | [11] |

Extra tests (Table B)

| **Virus** | **Standard PCR** | | **PCR with SYBR-Green** | **PCR with probe** | | **NESTED PCR** |
| --- | --- | --- | --- | --- | --- | --- |
| **Influenza C** |  | | [12] | [12] |  | [13] |
| **Influenza A H1N1pdm 2009** |  | | [10] | [10] with home-made probe | French National Reference Center System | home-made with primers from CDC sequencing and [14] |
| **Parainfluenzavirus** | Home-made system | [15] |  | Duplex home-made |  |  |
| **human Coronavirus** |  | |  | Duplex home-made |  | [16] |

**References**

1. Lu X, Chittaganpitch M, Olsen SJ, Mackay IM, Sloots TP, Fry AM, et al. Real-time PCR assays for detection of bocavirus in human specimens. J Clin Microbiol. 2006;44(9):3231-5. Epub 2006/09/07. doi: 44/9/3231 [pii] 10.1128/JCM.00889-06. PubMed PMID: 16954253; PubMed Central PMCID: PMC1594719.

2. Leruez-Ville M, Minard V, Lacaille F, Buzyn A, Abachin E, Blanche S, et al. Real-time blood plasma polymerase chain reaction for management of disseminated adenovirus infection. Clin Infect Dis. 2004;38(1):45-52. Epub 2003/12/18. doi: CID31470 [pii] 10.1086/380450. PubMed PMID: 14679447.

3. Heim A, Ebnet C, Harste G, Pring-Akerblom P. Rapid and quantitative detection of human adenovirus DNA by real-time PCR. J Med Virol. 2003;70(2):228-39. Epub 2003/04/16. doi: 10.1002/jmv.10382. PubMed PMID: 12696109.

4. van Elden LJ, van Loon AM, van der Beek A, Hendriksen KA, Hoepelman AI, van Kraaij MG, et al. Applicability of a real-time quantitative PCR assay for diagnosis of respiratory syncytial virus infection in immunocompromised adults. J Clin Microbiol. 2003;41(9):4378-81. Epub 2003/09/06. PubMed PMID: 12958272; PubMed Central PMCID: PMC193825.

5. Cane PA, Matthews DA, Pringle CR. Identification of variable domains of the attachment (G) protein of subgroup A respiratory syncytial viruses. J Gen Virol. 1991;72 ( Pt 9):2091-6. Epub 1991/09/01. PubMed PMID: 1895054.

6. Freymuth F, Eugene G, Vabret A, Petitjean J, Gennetay E, Brouard J, et al. Detection of respiratory syncytial virus by reverse transcription-PCR and hybridization with a DNA enzyme immunoassay. J Clin Microbiol. 1995;33(12):3352-5. Epub 1995/12/01. PubMed PMID: 8586738; PubMed Central PMCID: PMC228709.

7. Tapparel C, Cordey S, Van Belle S, Turin L, Lee WM, Regamey N, et al. New molecular detection tools adapted to emerging rhinoviruses and enteroviruses. J Clin Microbiol. 2009;47(6):1742-9. Epub 2009/04/03. doi: JCM.02339-08 [pii] 10.1128/JCM.02339-08. PubMed PMID: 19339471; PubMed Central PMCID: PMC2691104.

8. Petitjean J, Vabret A, Dina J, Gouarin S, Freymuth F. Development and evaluation of a real-time RT-PCR assay on the LightCycler for the rapid detection of enterovirus in cerebrospinal fluid specimens. J Clin Virol. 2006;35(3):278-84. Epub 2005/10/11. doi: S1386-6532(05)00243-X [pii] 10.1016/j.jcv.2005.09.006. PubMed PMID: 16214398.

9. Watkins-Riedel T, Woegerbauer M, Hollemann D, Hufnagl P. Rapid diagnosis of enterovirus infections by real-time PCR on the LightCycler using the TaqMan format. Diagn Microbiol Infect Dis. 2002;42(2):99-105. Epub 2002/02/23. doi: S0732889301003303 [pii]. PubMed PMID: 11858904.

10. van Elden LJ, Nijhuis M, Schipper P, Schuurman R, van Loon AM. Simultaneous detection of influenza viruses A and B using real-time quantitative PCR. J Clin Microbiol. 2001;39(1):196-200. Epub 2001/01/04. doi: 10.1128/JCM.39.1.196-200.2001. PubMed PMID: 11136770; PubMed Central PMCID: PMC87701.

11. Song MK, Chang J, Hong Y, Hong S, Kim SW. Direct multiplex reverse transcription-nested PCR detection of influenza viruses without RNA purification. J Microbiol Biotechnol. 2009;19(11):1470-4. Epub 2009/12/10. doi: JMB019-11-27 [pii]. PubMed PMID: 19996703.

12. Faux C. Influenza Type C. PCR for Clinical Microbiology: Springer Science+Business Media B.V; 2010. p. 311-2.

13. Matsuzaki Y, Abiko C, Mizuta K, Sugawara K, Takashita E, Muraki Y, et al. A nationwide epidemic of influenza C virus infection in Japan in 2004. J Clin Microbiol. 2007;45(3):783-8. Epub 2007/01/12. doi: JCM.01555-06 [pii] 10.1128/JCM.01555-06. PubMed PMID: 17215347; PubMed Central PMCID: PMC1829124.

14. Perez-Ruiz M, Pedrosa-Corral I, Sanbonmatsu-Gamez S, Navarro-Mari M. Laboratory detection of respiratory viruses by automated techniques. Open Virol J. 2012;6:151-9. Epub 2012/12/19. doi: 10.2174/1874357901206010151 TOVJ-6-151 [pii]. PubMed PMID: 23248735; PubMed Central PMCID: PMC3522051.

15. Bellau-Pujol S, Vabret A, Legrand L, Dina J, Gouarin S, Petitjean-Lecherbonnier J, et al. Development of three multiplex RT-PCR assays for the detection of 12 respiratory RNA viruses. J Virol Methods. 2005;126(1-2):53-63. Epub 2005/04/26. doi: S0166-0934(05)00035-2 [pii] 10.1016/j.jviromet.2005.01.020. PubMed PMID: 15847919.

16. de Souza Luna LK, Heiser V, Regamey N, Panning M, Drexler JF, Mulangu S, et al. Generic detection of coronaviruses and differentiation at the prototype strain level by reverse transcription-PCR and nonfluorescent low-density microarray. J Clin Microbiol. 2007;45(3):1049-52. Epub 2007/01/19. doi: JCM.02426-06 [pii] 10.1128/JCM.02426-06. PubMed PMID: 17229859; PubMed Central PMCID: PMC1829107.
