# Supplementary material for: Evaluation of Four Commercial Multiplex Molecular Tests for the Diagnosis of Acute Respiratory Infections
Source: PLoS One. 2015 Jun 24;10(6):e0130378. doi: 10.1371/journal.pone.0130378 (PMC4481272; doi:10.1371/journal.pone.0130378)
Supplement: S1 Table — (DOCX) [file pone.0130378.s002.docx]

**Table S1. Bacterial etiologies identified, co-infection with viruses and viral results.** (*Myco pneumo: Mycoplasma pneumoniae; Strepto pneumo: Streptococcus pneumoniae; Haemo inf sp.: Haemophilus influenzae species; Staph aur: Staphylococcus aureus; Morax catarr: Moraxella catarrhalis; Klebs pneumo: Klebsiella pneumoniae; Legio sp*.: *Legionella species;* Inf B: Influenza B; H1N1pdm09: Influenza A H1N1pdm 2009; PIV 2: Parainfluenzavirus 2; hRSV A, B: human Respiratory Syncitial Virus A, B; EV: Enterovirus; hRV: human Rhinovirus; hBoV: human Bocavirus; hCoV 229, OC43, NL63: human Coronavirus type 229E, OC43, NL63; hMPV A: human Metapneumovirus A).

| **Bacteria (Not confirmed results)** | **Nb** |
| --- | --- |
| **Myco pneu** | 6 |
| **Haemo inf sp.** | 4 |
| **Morax** | 20 |
| **klebsella pneumoniae** | 4 |
| **Staph aur** | 35 |
| **Strepto pneumo** | 28 |
| **Legio sp** | 7 |
| **Total number of bacteria detected** | 104 |
|  |  |
| **Morax catarr + Legio sp.** | 1 |
| **Staph aur + klebs pneumo** | 2 |
| **Staph aur + Legio sp.** | 1 |
| **Strepto pneumo + Morax catarr** | 5 |
| **Strepto pneumo + Staph aur** | 3 |
| **Myco pneu + Staph aur** | 1 |
| **Myco pneu + Strepto pneumo** | 1 |
| **Total number of bacterial dual infections** | 14 |
|  |  |
| **Strepto pneumo + Staph aur + Legio sp.** | 2 |
| **Strepto pneumo +Staph aur + Haemo inf sp.** | 1 |
| **Strepto pneumo + Staph aur +Myco pneumo** | 1 |
| **Strepto pneumo + Haemo inf sp. + Staph aur + Morax catarr** | 1 |
| **Strepto pneumo + Haemo inf sp. + Morax catarr** | 1 |
| **Total number of bacterial co-infections (at least 2 other bacteria)** | 6 |
|  |  |
| **Total number of bacteria positive samples** | 77 |
| **Percentage of bacteria positive samples (n=166)** | 25.30% |
|  |  |
| **EV / hRV + Staph aur + Legio sp.** | 1 |
| **EV / hRV + HboV + Strepto pneumo + Morax catarr** | 1 |
| **hCoV NL63 + Strepto pneumo + Morax catarr** | 1 |
| **hCoV NL63 + Strepto pneumo +Staph aur + Haemo inf sp.** | 1 |
| **H1N1pdm09 + Strepto pneumo + Staph aur** | 1 |
| **hMPV A + Strepto pneumo + Staph aur +Myco pneumo** | 1 |
| **Inf B + Staph aur + klebs pneumo** | 1 |
| **Inf B + Strepto pneumo + Staph aur** | 1 |
| **Inf B + Strepto pneumo + Haemo inf sp. + Staph aur + Morax catarr** | 1 |
| **hRSV B + Strepto pneumo + Myco pneumo** | 1 |
| **hRSV A + hMPV A + Strepto pneumo + Haemo inf sp. + Morax catarr** | 1 |
| **EV / hRV + Haemo inf sp.** | 1 |
| **EV / hRV + klebs pneumo** | 1 |
| **EV / hRV + Morax catarr** | 3 |
| **EV / hRV + Strepto pneumo** | 1 |
| **EV / hRV + hMPV A + Strepto pneumo** | 1 |
| **hBoV + Morax catarr** | 1 |
| **hCoV 229 + Morax catarr** | 1 |
| **hCoV NL63 + Morax catarr** | 1 |
| **hCoV NL63 + Strepto pneumo** | 1 |
| **hCoV OC43 + Myco pneumo** | 1 |
| **hCoV OC43 + Staph aur** | 1 |
| **hMPV A + Strepto pneumo** | 1 |
| **Inf B + Strepto pneumo** | 1 |
| **H1N1pdm09 + Myco pneu** | 1 |
| **H1N1pdm09 + Morax catarr** | 2 |
| **H1N1pdm09 + Staph aur** | 2 |
| **H1N1pdm09 + Strepto pneumo** | 1 |
| **hRV + Strepto pneumo** | 2 |
| **hRV + Staph aur** | 2 |
| **Inf B + Staph aur** | 1 |
| **Inf B + hBoV + Staph aur** | 1 |
| **hRSV B + AdV + Strepto pneumo** | 1 |
| **PIV 2 + Strepto pneumo** | 1 |
| **Total number of co-infections (Virus/bacteria)** | 40 |
|  |  |
| **Viruses (Confirmed results)** | **Nb** |
| **Total number of virus detected** | 95 |
| **Total number of viral dual infections** | 8 |
| **Total number of viral positive samples** | 87 |
| **Percentage of viral positive samples (n=166)** | 52.40% |
|  |  |
| **Total number of positive samples (confirmed for viruses but not for bacteria)** | 124 |
| **Percentage of positive samples (n=166)** | 74.70% |
