# Supplementary figures and images for: Evaluation of Four Commercial Multiplex Molecular Tests for the Diagnosis of Acute Respiratory Infections
Source: PLoS One. 2015 Jun 24;10(6):e0130378. doi: 10.1371/journal.pone.0130378 (PMC4481272; doi:10.1371/journal.pone.0130378)

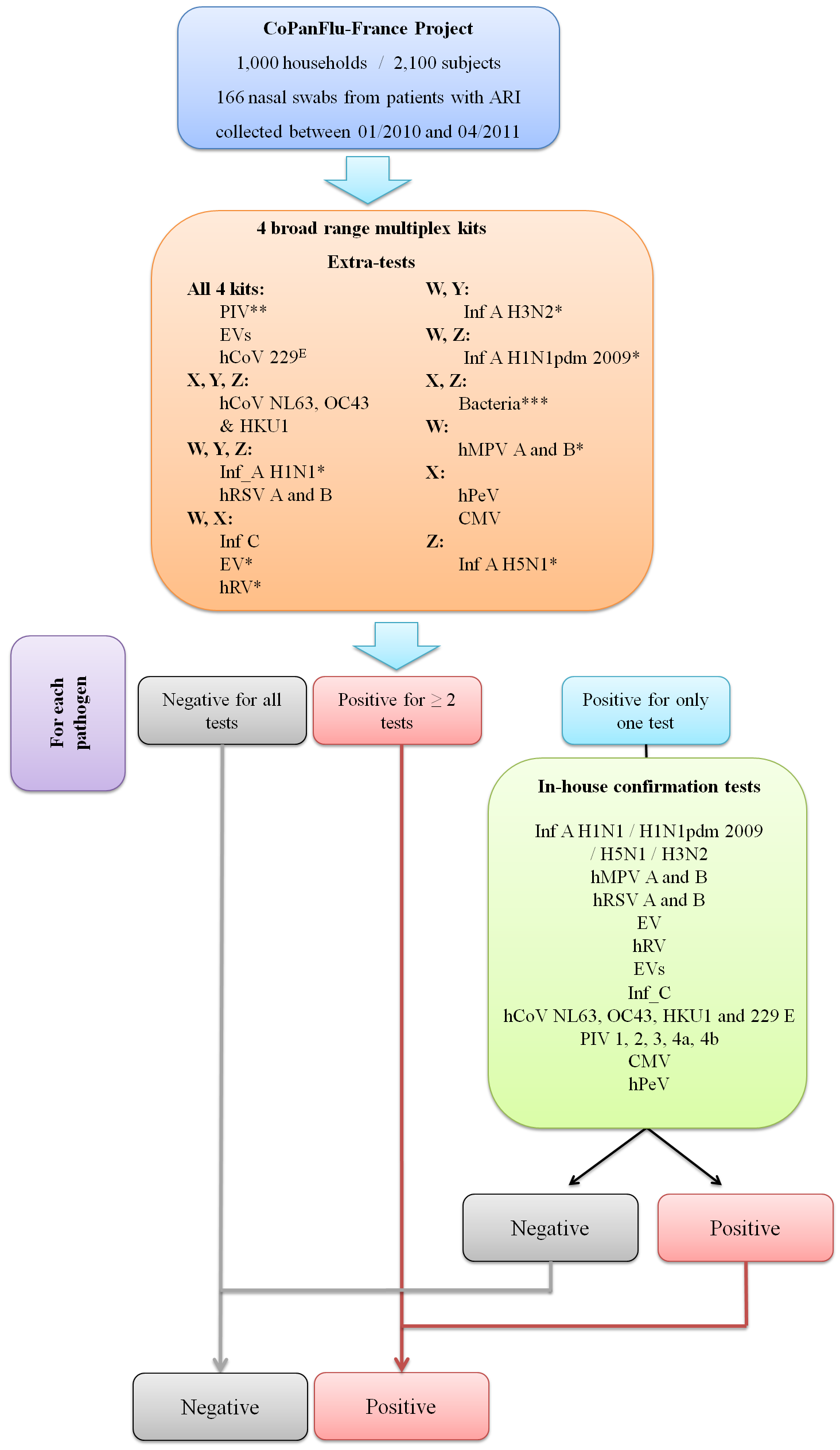

Supplement: S1 Fig — (TIFF) [file pone.0130378.s003.tiff]
